# Exploring the Intersection Between Academic and Professional Practice During the COVID-19 Pandemic: Undergraduate and Graduate Nursing Students’ Experiences

**DOI:** 10.1177/08445621211037147

**Published:** 2022-09

**Authors:** Sherry Espin, Karen LeGrow, Sue Bookey-Bassett, Donald Rose, Elaine Santa Mina, Alyssa Indar

**Affiliations:** 1 Daphne Cockwell School of Nursing, 7984 Ryerson University, Toronto, ON, Canada; 2 70373 Faculty of Health Sciences and Wellness, 10025 Humber College, Toronto, ON, Canada

**Keywords:** Qualitative description, nursing students, COVID-19, aesthetic reflections, practice development

## Abstract

**Background:**

The coronavirus disease-2019 (COVID-19) pandemic has implications for students who are also nurses.

**Purpose and Methods:**

This qualitative descriptive study used a practice development approach to explore the intersection between academic and professional work experiences for undergraduate Post-Diploma Registered Practical Nurses bridging to Registered Nurse Bachelor of Science in Nursing students and Master of Nursing graduate nursing students during the first wave of the COVID-19 pandemic. The study incorporated critical aesthetic reflections that focused on the personal and aesthetic ways of knowing, as a data collection approach and knowledge dissemination strategy.

**Results:**

Analysis of the narrative component of participants’ reflections revealed the following themes: sensing a “call to duty,” experiencing a myriad of emotions, shifting societal and individual perceptions of nursing, and learning in an uncertain environment.

**Conclusions:**

The results of the study can inform educational strategies and academic policies to support this unique nursing population, who are frontline practitioners as well as student learners.

## Background

Given the continual development of new therapies, technologies, and innovations, nurses are constantly advancing their knowledge, skills, and leadership abilities to meet ever-changing dynamic challenges that arise within our health care system. One way in which nurses meet these challenges is to pursue higher, post-secondary nursing education. Nurses at different professional levels seek educational opportunities to obtain advanced credentialing; Registered Practical Nurses (RPNs) work toward a Bachelor of Science in Nursing degree (BScN), while BScN Registered Nurses (RNs) study to attain a Master of Nursing degree (MN). To achieve these academic and professional advancements, RPNs and RNs often maintain full or part-time employment while in pursuit of advanced nursing education.

However, the coronavirus disease-2019 (COVID-19) pandemic continues to present serious implications for nurses who are also students. According to Ontario provincial modeling for COVID-19 testing, the pandemic will continue until the Ontario population vaccination has reached a critical mass to achieve herd immunity. As a result, this extended trajectory creates unusual challenges and barriers for nursing students who are in the process of completing their respective nursing programs, while simultaneously maintaining their clinical practice. Consequently, learning has moved from exclusively face-to-face in class instruction to synchronous and asynchronous online learning, and clinical placements have been fundamentally modified or cancelled.

The global pandemic affects nurses personally, socially, and in the context of the community in which they work. Recent work highlights that undergraduate nursing students feel increased levels of stress and may require additional support to improve knowledge and coping strategies to prepare them for work during a pandemic and post pandemic (Aslan & Pekince, 2020; Goni-Fuste et al., 2021; Heilferty et al., 2021; Usher et al., 2020).

The intersection between their professional obligations, in their role as a nurse, and their academic obligations, in their role as a student, is unique. Hence, there is a paucity of literature describing the intersection between student (undergraduate and graduate) and health care professional roles during a global health crisis. Transitioning from practice into the dual, simultaneous roles of a student and practitioner can be challenging and requires the continuous support of faculty and clinical placement preceptors (Cruz et al., 2017; Suva et al., 2015). Research is needed to understand the additional challenge of navigating this role intersection during an unprecedented global pandemic, and to foster the development of innovative education approaches and strategies that support learning in times of societal crises.

The purpose of this study was to explore the COVID-19 pandemic impact on education, learning, and professional work of Canadian Nursing School’s undergraduate and graduate students. Specifically, the competing academic-professional employment obligations, which arise with the disruption of academic classes and clinical placements while employed and practicing as a nurse during a pandemic crisis are described.

This qualitative descriptive study used a practice development (PD) approach to explore the intersection between academic and professional work expectations, as experienced by undergraduate Post-Diploma RPNs bridging to RN BScN students and RN BScN to MN graduate nursing students during the first wave of the COVID-19 global pandemic.

## Theoretical orientation

PD is an engaging and innovative methodology to promote person-centered cultures, actively, within academic and health care settings (Manley et al., 2008; McCormack & McCance, 2017; McCormack et al., 2013; Titchen & McCormack, 2010). Central to PD is a foundation of active learning in which leaders engage authentically with students and practicing nurses and teams to blend personal qualities; and critical and creative methods that invite open dialog with self to learn from practice skills and wisdom (Dewing, 2010; Manley et al., 2008; McCormack et al., 1999, 2009; Titchen & McCormack, 2010). This unique approach encourages practicing nurses and students to build clinical and learning environments that promote growth and personal/professional transformation.

There is a growing body of evidence that uses this strategy in academic settings to support practice-based students in the development of critical thinking skills from practice contexts, through creative methods (Indar et al., 2018; LeGrow & Espin, 2017; LeGrow et al., 2016). The creative active learning activity used in this study is intended to help students consolidate their thinking about their experience during the COVID-19 pandemic at the simultaneous role intersection of student and practicing nurse. We aim to incorporate this innovative, creative, and meaningful approach within nursing education to promote reflective practice and its influence on professional development, within practice-based education programs.

## Methods

This qualitative descriptive study (Sandelowski, 2010) explored the intersection between academic and professional work experiences for undergraduate Post-Diploma Degree Program (PDDP) RPNs bridging to RN BScN students and RN BScN nursing students completing a MN graduate degree during the COVID-19 pandemic. The study received ethics approval from the academic institution's Research Ethics Board. This study was conducted during the first wave of the COVID-19 pandemic, from March 2020 to June 2020.

### Participants

Participants were recruited from 150 PDDP (BScN) and 33 graduate level Master of Nursing Degree Program (MN) nursing students who were enrolled in a clinical placement during the Winter 2020 semester. Following grade submission by faculty for the respective courses (to protect against potential participant perception of coercion), email invitations were distributed to prospective student participants via their respective program coordinators, inviting them to participate in the study.

Of the 16 study participants, 11 were recruited from the PDDP program and five were recruited from the MN program. Participant data were collected regarding role (e.g., RN or RPN), current work status (e.g., full-time, part-time, and casual), number of years of experience in nursing work and current work setting (see Tables 1 and 2).

**Table 1.** Data From MN Participants.

	Current role	Work status	Years of experience	Work setting
P1	RN	Casual	3	Acute Medicine/Surgery
P2	RN	PT (3 positions)	4	Acute Medicine; Occupational Health
P3	RN	FT	8	Acute Care—ICU/CCU
P4	RN	PT	6	Acute Care—ICU/CCU
P5	RN	PT	4.5	Acute Care—Emergency Department

*Note.* MN = Master of Nursing; RN = Registered Nurse; PT = part time; FT = full time.

**Table 2.** Data From PDDP Participants.

	Current role	Work status	Years of experience	Work setting
P1	RPN	Casual	3	Long-term Care Facility
P2	RPN	PT	2	Long-term Care Facility
P3	RPN	FT	10	Acute Care— Surgery
P4	RPN	Unemployed	5	N/A
P5	RPN	Unemployed	6	N/A
P6	Graduate of RPN program	Working Outside of Health Care Setting	None	N/A
P7	RPN	FT	8	Addiction & Mental Health/Psychiatric Hospital
P8	RPN	FT	2	Addiction & Mental Health/Psychiatric Hospital
P9	RPN	Casual	1	Long-term Care Facility
P10	RPN	PT	3	Long-term Care Facility
P11	RPN	Casual	1	Public Health Unit

*Note.* RPN = Registered Practical Nurse; PDDP = Post-Diploma Degree Program; FT = full-time; PT = part time.

### Data collection

A PD approach, using creative, active-learning strategies, informed the critical reflection activity, which resulted in the creation and collection of various aesthetic artifacts. Data collection occurred during May and June 2020. Prospective participants were sent a link via their school email address to complete a secure Google form. The first section of this form collected demographic information, reflected in Tables 1 and 2. The second section contained four open-ended questions (see Table 3) intended to help participants reflect on the experience of being a nurse and student during the Winter 2020 semester, which coincided with the first wave of the COVID-19 pandemic.

**Table 3.** Reflective Questions.

1.	What it is like for you to be an academic student and a professional nurse role at the same time, during the COVID-19 pandemic?
2.	How has COVID-19 impacted your learning and education experience?
3.	How do you perceive or experience equity in your role as a student and a nurse?
4.	How has COVID-19 shaped your thinking about the nursing profession?

Using arts-based knowledge translation dissemination strategies (Parsons & Boydell, 2012), a website (aesthetic-reflection.com) was created, post study, to showcase these aesthetic artifacts, rather than analyze them. The student aesthetic artifacts included poems, music, paintings, photographs/pictures, and so on. A more detailed description of the process will be available in future publications. Following the construction of the aesthetic artifacts, participants were asked to complete an online Google form of open-ended questions to reflect on the simultaneous role experiences of nurse and student during the first wave of the COVID-19 pandemic.

### Data analysis

Demographic data were analyzed using descriptive statistics. Narrative data from the student responses were analyzed using a thematic qualitative approach (Green & Thorogood, 2018). Consistent with this approach, all members of the research team were immersed in the narrative data via in-depth reading and coding of the narrative responses. All team members were present at the data analysis meeting, given the diversity of perspectives and experiences within nursing education and research. Coded responses were collected and used to construct a series of codebooks. The research team met at regular intervals to discuss the organization of the codebooks, which led to the construction of the themes. Several strategies were used to enhance the rigor or trustworthiness, as originally outlined by Lincoln and Guba (1985) (Schwandt et al., 2007). Our team engaged with the data for a prolonged period, to develop a deep and nuanced understanding of the phenomenon. Our analytic meetings included all team members for the purpose of promoting diverse and critical dialog and all decisions were recorded to keep a clear audit trail.

## Results

Themes that were revealed through the analysis of the cohort data from MN and PDDP narratives include: (1) sensing a “call to duty”; (2) experiencing a myriad of emotions; (3) shifting societal and individual perceptions of nursing; and (4) learning in an uncertain environment.

### Theme 1: Sensing a “Call to Duty”

As professional nurses and students, many participants described the notion of being “called to duty” to fulfill their critical nursing role to care for patients, and to support one another during the pandemic:

As an ICU nurse, I have witnessed the horrible effects of this virus and its ability to kill people. ICU nurses right now are force multipliers and my commitment is within my call to duty and my obligation to fight this virus. Not only is my presence important within the presentation, it is also extremely important for other nurses that need support during this time where we are faced with a lack of staff, support and supplies during a global wide pandemic. (N1, MN—P12)

Other participants shared this same sense of obligation to the duty of nursing as a priority, with the student role “on hold”:

As with our nature of why we enter into nursing in the first place, we all discussed the internal “pull” of performing as an essential worker in our frontline jobs for COVID-19 (N1, MN—P8).

Being the frontlines, it felt almost that we were the military troops that were sent off to an unprecedented war, where my education could have been placed on halt in order to meet the needs of the health care system. (N1, MN—P15)

Participants also reflected that this “call to duty” was not without risk, noting their personal sacrifices to protect their families while providing care to those in need:

Responsible for my grandparents, both of whom have medical conditions placing them at high risk. I was forced to make a difficult decision between keeping my family safe and fulfilling my obligation as a nurse. (N1, PDDP—P6)

### Theme 2: Experiencing a Myriad of Emotions

Participants described feeling a myriad of emotions during the pandemic. They reported feeling “stressed” and “exhausted” by the increasing demands and challenges presented in the clinical environments. As one participant stated

We were all fearful and conveyed thoughts about being mentally, physically, and emotionally exhausted. [We] faced a new norm of pulling double shifts or having to isolate from our families and loved ones. Naturally, one can understand the fatigue and stress faced in being a professional frontline nurse during COVID. (N1, MN—P8)

Despite these challenges, other participants described their feelings of pride in the nursing profession, as well as hope and gratitude:

I think the nursing profession has a huge heart. The morals amongst us are super strong. We are fighting an invisible war together. (N4, MN—P16)

In addition, feelings of loss were noted among participants, primarily in relation to their academic experience. Participants shared their sense of losing the opportunity to engage in the traditional end-of-program celebrations:

COVID stripped me of the sense of celebration I am supposed to feel when I complete my Masters. The happiness I was supposed to feel in presenting at the symposium and the happiness I would have felt congratulating my peers on finishing theirs. (N2, MN—P12)

Furthermore, participants noted that the COVID-19 pandemic created a time of uncertainty and fear. Participants expressed feeling uncertain about how they would complete their academic programs and whether they would be able to transition to their new roles as RNs. Participants also noted the responsibility to fulfill their obligations as a nurse, as reflected in this statement:

When placement and classes were cancelled due to the COVID-19 pandemic, there was a lot of uncertainty with how my remaining month of the Post-Diploma program would play out. After six years of nursing school, I started to feel panic at the possibility of delaying graduation and my career. As well, being an academic student, close to graduating as an RN, put pressure on working immediately. Without even booking a date for the NCLEX, agencies were searching for new graduates. The confusion of how the semester would play out, coupled with the lack of easing into my RN role, was scary. (N1, PDDP - P6)

Others expressed the stress related to delays in completing the academic requirements necessary to graduate and obtain an RN license:

It is stressful having to finish courses and do supplementary work in order to meet clinical requirements while also needing to be available to work. There were delays in sending marks to the CNO so that I could get a temporary license which results in me having to work for less pay as an RPN when I could work as a temp RN right now and better support the needs of my hospital. (N1, PDDP—P10)

### Theme 3: Shifting Societal and Individual Perceptions of Nursing

Participants acknowledged a shift in public/societal perceptions of nursing, prompted by media coverage during the pandemic. While participants were pleased to see an increase in recognition of nurses’ contributions during COVID-19, they also indicated disappointment that it took a pandemic for the value of nurses to be truly appreciated:

I think after COVID-19 nursing will truly never be the same. Even after going to work, showering, and coming home – I have friends that I have not seen and frankly, do not want to hang out. I am most shocked by the number of individuals in society who are praising nurses for all of their hard work during COVID-19. I actually find it a bit degrading that I was never valued before “as a hero.” (N4, MN—P8)

Participants also described individual/personal realizations of weaknesses within the health care system. These included the perception of siloed work environments and the lack of essential resources to ensure staff safety.

COVID-19 has made me realize the silos that we work in. There are so many issues in the health care system that have only been magnified as a result of this pandemic. (N4, MN—P12)

Participants expressed a shift in perspective or appreciation of nursing as a profession and as a career option. Participants reflected on their recognition of the importance of nurses within health care teams.

This COVID-19 experience has shaped my thinking about the nursing profession because I learned to appreciate the importance of nursing and the interprofessional members in mitigating the COVID-19 pandemic … I realized the importance of various personnel from ancillary care providers to administrators in performing their respective tasks to facilitate in mitigating COVID-19. Ancillary care providers worked with the interprofessional team to disinfect potentially COVID-19 areas. Managers helped mitigate the contact of care providers from COVID-19. While nurses [were] working with patients and residents in the direct care of their health. As such, a collaborative team environment was of importance in shaping my perception of the nursing profession. (N4, PDDP—P2)

Other participants spoke about how their perspectives changed, related to the realities of nursing practice during a pandemic:

COVID-19 has taught me that as a nurse, we should be ready for anything. Some people at my work talk about how they are working again with COVID-19 after working through SARS back then. I think that the importance of the nursing profession won’t go away. I am glad that I chose the nursing profession. (N4, PDDP—P3)

### Theme 4: Learning in an Uncertain Environment

Participants shared differing perspectives related to their academic experiences. MN graduate students described support from nursing faculty as a key factor that enhanced their ability to balance their concomitant academic and professional expectations. PDDP undergraduate students perceived their academic experiences differently primarily due to their loss of clinical placement experiences.

Graduate program faculty were described as accommodating and flexible in enabling students to complete academic requirements, in the context of the impact of COVID-19 on academic settings.

The MN professors and directors were lenient and understanding … I felt this strongly impacted all students’ abilities to finish the program successfully. In particular, we were given a choice about a final grade and project. Having been given a choice, rather than told what the game plan is, plays a big role in satisfaction, resiliency, and ability to function effectively within the program while facing COVID-19. (N2, MN—P8)

MN participants also described instances in which the pandemic provided real-time opportunities to apply or consolidate high-level skills learned in the MN program, as helpful in their current and future roles and practice:

It is rare that we as learners, in a very novice role, have a profound opportunity to become a change agent in less than four months. It is rare to be a part of such a change that will affect learning and practice for years to come. In a profession that has long been oppressed for years, any of these victories that nurses make in itself is an honour and deserves to be shared with the world. (N1, MN—P12)

However, PDDP students described the great impact on the cancellation of clinical placement and transition to online learning. Participants expressed a loss of face-to-face opportunities to learn from preceptors in the practice setting and having to develop new skills for online learning.

The COVID-19 pandemic has truly impacted my nursing practice and learning, especially the early end to clinical placements. I felt very fortunate to be placed on the General Internal Medicine, Geriatric and Stroke Unit for my nursing consolidation, especially since it provided me with hands-on learning experience in an acute hospital setting. Unfortunately, this opportunity ended earlier than expected. The learning gained from reading textbooks, watching videos, or practicing in a simulation lab is much different than the teaching provided directly from the preceptor or other nurses on the unit … I lost the opportunity to continue learning from an excellent preceptor on a unit that provided care for such a diverse and complex population, including the homeless and marginally-housed, mental health, addictions, stroke, cancer, infectious diseases, and other medical conditions. (N2, PDDP—P5)

Other participants described being upset due to the cancellation of clinical placements, its hindrance on their academic performance, and their feelings about that loss.

The pandemic led to a lot of changes in my academic settings. Placement was no longer occurring and all classes and exams were moved to an online format. Although this freed up more time for myself to work or rest more, I felt it did hinder my academics slightly as in-person lecture formats as well as test taking in a paper format is more beneficial to my learning as opposed to an online format. (N1, PDDP - P14)

## Discussion

The thematic descriptions of the MN graduate students and PDDP undergraduate students elucidated their experiences of the intersection between their dual roles as a practicing nurse and as a student. Although similar to research on the nursing student experience during the COVID-19 pandemic, the findings of this study provide a nuanced perspective of students in dual roles. Participants expressed a “call to duty” as one of four main themes, describing their experience of the intersection of these roles. Participants used analogies of war to describe their experiences and being called to serve on the frontline, even to the extent that their education could be “put on hold” while they served society. The sense of working for a greater good, and the expression of a “call-to-duty,” harkens back to a time during the First World War when Canadian nurses left their families to respond to the “call to duty” (Government of Canada, 2020). Their contribution to the war drew on their strengths and knowledge, their dedication to their work and, most importantly, to their patients (Government of Canada, 2020). Similar to the findings in this study, Dewart et al. (2020) observed the ongoing commitment, perseverance, and loyalty that nurses have consistently made during the pandemic and that students’ biggest concern during COVID-19 was the health of their patients and communities.

Participants in this study reported experiencing a myriad of emotions, namely, stress, fear, loss, and pride. Our findings are consistent with other studies that report that undergraduate nursing students experience stress related to COVID-19 (Aslan & Pekince, 2020; Heilferty et al., 2021; Lovrić et al., 2020). Sources of stress include moving from in-person to online learning, cancellation of clinical placements, watching the news, and worrying about the risk of infection. While much of the literature reports on undergraduate nursing students’ experiences during COVID-19, the current study found graduate students also experience stress and fear related to balancing the responsibilities of an advanced role with their family commitments.

The fear experienced by students in the PDDP and MN programs is similar to findings in other studies, in which students report a fear of getting sick, a fear of uncertainty, a fear that the disease will affect one's family, and generally feeling of being unsafe in one's community (Aslan & Pekince, 2020; Lovrić et al., 2020).

Participants in this study reported a sense of loss as they missed the traditional celebrations of achievement, upon completion of their education program. As noted by D’Aquin (2020), the end of the semester should be a time of celebration and setting new goals for one's career; but the pandemic created uncertainty in one's desired career trajectory. Similarly, post-licensure baccalaureate nursing students also raised concerns about the meaning of interruption in their nursing education for their future careers as RNs (Dewart et al., 2020).

Participants in this study also acknowledged a sense of pride, hope, and gratitude for the nursing profession. Dewart et al. (2020) suggested that a sense of pride comes from recognizing the substantial dedication, contributions, and sacrifices made by nurses during the pandemic, who consistently prioritize the needs of patients and communities. In addition, Heilferty et al. (2021) reported that undergraduate students expressed positive experiences and recognition of lessons learned.

Study participants described the changing societal perceptions of the nature and value of nursing work that may have been influenced by media coverage of nursing during the pandemic. A body of literature describes the historical and contemporary portrayal of nurses as moral figures (Price & McGillis-Hall, 2014). There is discourse that critiques these images for their minimizing perception of nurses as skilled, knowledgeable workers (Gordon & Nelson, 2005). The participants in this study highlighted the inconsistency of public perceptions of nurses and the treatment of nurses during the COVID-19 pandemic. For example, across the trajectory of the first wave, nursing work has been praised and nursing actions have been viewed as heroic, which may have paradoxical long-term negative consequences for the nursing profession (Hennekam et al., 2020; McAllister et al., 2020; Stokes-Parish et al., 2020). Additionally, local nursing organizations have highlighted that nurses’ demands for decreased workplace risks have been insufficiently addressed (RNAO, 2020).

The intersectionality of roles during COVID-19 impacted the academic experience of the participant groups differently, reflecting their unique positions in the nursing education process, and also their unique professional development activities and career trajectories. Faculty support to assist students to balance academic and employment commitments was evident. While there were expressions of loss of clinical practice hours and resultant opportunities for continued project leadership for the MN graduate student participants, they clearly described their ability to apply their graduate level competencies to inform their practice requirements during this uncertain period. The MN graduate students reported that the COVID-19 pandemic provided an opportunity to reach beyond their own personal and professional needs and respond at the organizational and environmental levels, which are hallmarks of advanced practice nursing education (CNA, 2019; Hamric et al., 2014; Staples et al., 2016).

For the PDDP participants, the loss of clinical practice was challenging, as it interfered with developing a sense of competency and familiarity with the RN role. The PDDP students valued point-of-care placement opportunities to learn practice expectations and to become socialized into the RN degree prepared role. Further, the PDDP placements were direct point-of-care; whereas the MN practicum placements focused on developing advanced competencies in the domains of leadership and education consistent with an advanced practice role and was not solely focused on providing direct care. The transition to online learning and lectures was perceived as a hindrance by many PDDP learners. For example, PDDP participants expressed frustration in balancing the professional role with academic expectations especially with the need for academic eligibility requirements for processing RN registration. These issues were not as apparent within the MN participant cohort.

## Limitations

The exploratory nature of this study provided an opportunity to learn about the experiences of nursing students in the dual role of student and practitioner during the COVID-19 pandemic. Given the needs of the participants, data collection methods were constructed in a way to minimize contact with the research team and facilitate study participation in a flexible manner. The convenience of this approach was necessary given the implications of the COVID-19 pandemic but did not allow for further probing of responses. In addition, this was a single site research study.

## Implications

The findings from this research study have implications for nursing education, research, and practice. Understanding the experiences of students in dual roles is particularly important during acutely stressful times, such as the COVID-19 pandemic. Educators play a critical role in supporting students by providing flexible academic policies and teaching–learning methods. Further research is needed to discover best practices to support students in dual roles, particularly as the impact of the COVID-19 pandemic evolves. Practice leaders and administrators can also positively enhance clinical environments that support learning for nursing students during uncertain times through flexible, responsive scheduling, assignments, and expectations to help balance nurses’ professional and academic responsibilities. These implications are critical to ensure that nurses remain in their essential practice roles, as well as continue advanced credentialing and education, in order to meet our country's pressing health care requirements.

## Conclusion

This study incorporated a critical reflection activity that provided participants with an opportunity to capture their thoughts, feelings, and emotions, during the first wave of the COVID-19 pandemic. This critical reflective process was a strategy used to help students critically consolidate and creatively describe what they have experienced related to the intersection of their dual roles as students and practicing nurses during the COVID-19 pandemic. The narrative accounts of their experiences highlight their collective voices. The study findings provide a starting point to build current and future intentional approaches to mitigate educational and practice issues that arose during the pandemic and that may arise in future pandemics. Such intentional approaches include pedagogical delivery options, course sequencing, and strategies, supports, and academic and/or clinical policies and procedures to support this unique nursing population at the intersection of their learning, as students and their practice as frontline practitioners.
